# Perceived Stress in Dentists and Dental Students of Latin America and the Caribbean during the Mandatory Social Isolation Measures for the COVID-19 Pandemic: A Cross-Sectional Study

**DOI:** 10.3390/ijerph18115889

**Published:** 2021-05-30

**Authors:** Roberto A. León-Manco, Andrés A. Agudelo-Suárez, Ana Armas-Vega, Márcia Cançado Figueiredo, Francisca Verdugo-Paiva, Yrma Santana-Pérez, Andrés Viteri-García

**Affiliations:** 1 Faculty of Dentistry, Cayetano Heredia Peruvian University, Lima 15102, Peru; roberto.leon@upch.pe; 2 Faculty of Dentistry, University of Antioquia, Medellín 050010, Colombia; 3 Faculty of Dentistry, Central University of Ecuador, Quito 170102, Ecuador; acarmas@uce.edu.ec; 4 Faculty of Dentistry, Federal University of Rio Grande do Sul (UFRGS), Porto Alegre 90035-003, Brazil; mcf1958@gmail.com; 5 UC Evidence Center, Cochrane Chile Associated Center, Pontificia Universidad Católica de Chile, Santiago 8331150, Chile; francisca.verdugo@uc.cl; 6 Epistemonikos Foundation, Santiago 7510321, Chile; andres.viteri@ute.edu.ec; 7 Faculty of Dentistry, University of Zulia, Maracaibo 4011, Venezuela; yrmasantana@gmail.com; 8 Center for Research in Public Health and Clinical Epidemiology (CISPEC), Faculty of Health Sciences Eugenio Espejo, UTE University, Quito 170508, Ecuador

**Keywords:** stress, psychological, dentists, students, dental, COVID-19, health surveys

## Abstract

This study aims to determine the impact of the COVID-19 pandemic, specifically considering the mandatory social isolation measures implemented, on the perceived stress of a sample of dentists and dental students from Latin America and the Caribbean, as well as the associated sociodemographic and pandemic-related variables. A cross-sectional survey was conducted with a sample of 2036 dentists and dental students (1433 women). For the main outcome, the 14-item Perceived Stress Scale (PSS-14) was used. The survey also questioned sociodemographic aspects, questions on the COVID-19 pandemic, health variables, and habits. Descriptive, bivariate, and multivariate analyses (linear regression) were applied to observe the factors associated with perceived stress. The PSS-14 mean score was 24.76 (±11.76). Hierarchical regression models showed significant variables associated with the PSS-14 scores: income level during mandatory social isolation, having older adults under care during mandatory social isolation, self-perceived level of concern regarding COVID-19, self-perceived health, Coffee consumption during mandatory social isolation. In general terms, the pandemic has influenced the personal, social, labor, and everyday life of dental staff and affected the mental health of this population specifically when perceived stress is considered. Public policies, strategies, and mental health surveillance systems are required for this population.

## 1. Introduction

The SARS-CoV-2 outbreak has established a new order at the global level, considering its epidemiological and biological characteristics (including its high infectivity rate and daily death count) [1]. The epidemiological behavior in the countries has been different—although, in general terms, a rapid spread has been observed. Specifically, in Latin America, mathematical and statistical investigations involving the effective reproductive number (a parameter used to follow up on an epidemic) reveal a value >2 for 2019, indicating exponential growth [2]. Since the WHO declared the coronavirus disease 2019 (COVID-19) outbreak a pandemic, governments have established escalating containment measures (such as border closures, lockdowns, and other isolation measures) to reduce viral transmission [3].

In the context of the COVID-19 pandemic, it seems important to pay attention to the possible risks that dentists may be subjected to during their practice [4,5]. The scientific literature has drawn attention to factors such as the close contact (face to face) with patients, which causes constant exposure to fluids such as saliva and blood, among others; the spread of aerosols during dental procedures; and the possible spread of viral agents in the air. Moreover, the persistence of the viral agent in operating rooms should be considered [4,5]. However, some studies have mentioned that adherence to biosafety protocols by dental health professionals is high [6]. The relationship of other variables and conditions related to the pandemic cannot be ignored, among which could be mentioned the level of knowledge about COVID-19, preventive behaviors and practices as well as the availability of training courses for the management of this pandemic in dentist and dental students [7,8,9].

In general terms, dental practice has undergone numerous changes during the pandemic, specifically considering lockdowns and isolation measures. Studies conducted globally involving dental staff show that this situation has had a significant impact on their personal life, financial status, and the prospects of their professional career [9,10,11,12]. Similarly, other effects of the pandemic including the field of nutrition (a possible increase of overweight and obesity) [13], changes in family dynamics by the presence of children, or elders that represent some burden when they are economic providers, household income, among others [9,10]. When mental health is considered, several studies have been conducted to evaluate the impact of the pandemic on perceived stress, anxiety, depression, and other mental health indicators in dentists, dental students, and support personnel [14,15,16,17,18,19,20,21,22]. In addition, some studies have investigated the consequences of the national lockdowns on the perceived distress in these dental workers and other impacts [23].

In Latin America, government actions to deal with the COVID-19 pandemic have not been implemented in a unified manner [24]. In addition, the issues caused by this pandemic could have been amplified by strong, structural social inequalities, which constitute an important obstacle for the resolution of important challenges in the region. Moreover, the significant limitation of clinical and surgical activities in the dental sector has represented a very impactful measure on its economy [25]. Nevertheless, the restriction for dental practice has not been implemented equally in all Latin America and the Caribbean countries. As far as we know, in most countries, the dental practice was limited to urgency and emergency treatments which could not be postponed -mainly in charge of the public sector- [8], but in other, the dental practice was carried out -although diminished substantially-, by implementing biosafety protocols. From this perspective, it seems appropriate to explore the impact of the pandemic on the general situation of dental health workers in this territory. This information constitutes an input for the implementation of global policies and strategies that contribute to the wellbeing of these personnel. It is highlighted that, during the measures of social isolation, the majority of government aid was focused on first-line health professionals [26].

Research focused on mental health topics related to the pandemic among dental staff in Latin America and the Caribbean is scarce. A scientific literature exploration reveals some published studies and gray literature carried out in specific settings and with specific samples [27,28], as well as multicenter studies in dental students in Latin American countries (Brazil and Chile) [29]. Hence, the extent of scientific research in this region should be increased through studies with methodologies targeted at specific problems and by analyzing the determining factors of physical, mental, and psychosocial health indicators. Accordingly, this study aims to: (1) determine the impact of the COVID-19 pandemic, specifically considering the social isolation measures implemented, in the perceived stress of a sample of dentists and dental students from Latin America and Caribbean, and (2) analyze the association of the perceived stress with sociodemographic and other pandemic-related variables in the study population. 

## 2. Materials and Methods

### 2.1. Design, Data Collection, and Setting

This manuscript was written according to the STROBE guidelines for observational studies [30]. A cross-sectional study was conducted by means of an anonymous survey administered online to a convenience sample of dentists and dental students in 21 Latin American and Caribbean countries (Argentina, Bolivia, Brazil, Chile, Colombia, Costa Rica, Dominica, Ecuador, El Salvador, Granada, Guatemala, Honduras, Mexico, Nicaragua, Panama, Paraguay, Peru, Puerto Rico, the Dominican Republic, Uruguay, and Venezuela). The initial sample of respondents was 2442.

A questionnaire was designed using the Google Forms platform. A pilot test was carried out with a sample of 30 participants to improve intelligibility and assess time to completion and internal consistency (understanding of the diverse questions of the instrument for gathering information). Fieldwork took place from 15 May to 26 August 2020. The online questionnaire was distributed through digital media: Facebook groups, WhatsApp messages, emails, and institutional invitations to different dental schools. In addition, snowball techniques were applied to increase the number of participants. The questionnaire gathered information about sociodemographic data and included questions about the COVID-19 pandemic (knowledge and practices), self-perceived mental and physical health, and social support. Surveys with errors in the information record were excluded, and the final sample of respondents was 2036 (cooperation rate: 83.4%).

### 2.2. Variable Definitions

For the purposes of this study, the following variables were considered:

#### 2.2.1. Dependent Variable

This is the main outcome of the analysis. 14-item Perceived Stress Scale (PSS-14) was applied to measure stress in the participants [31]. This instrument comprises 14 items: 7 positive items and 7 negative items rated on a five-point Likert scale (0 = Not at all/Never, 1 = Rarely, 2 = Sometimes, 3 = Fairly often, and 4 = Very often). The positively worded items were reverse-scored prior to the analysis. All items were added, and the total score ranged from 0 to 56; the higher the score, the greater the self-perceived stress.

#### 2.2.2. Independent Variables

- Sociodemographic variables including: sex, age, type of academic training, specialty (field of postgraduate study), place of origin, the number of people at home during mandatory social isolation, children and older adults under care during mandatory social isolation; labor, academic, domestic (home) responsibilities during mandatory social isolation; income level during mandatory social isolation.
- Health variables including: The body mass index (BMI), which is defined as a person’s weight in kilograms divided by the square of their height in meters (kg/m^2^). With this information, and according to the parameters of the WHO [32], the following characteristics were determined: (a) underweight: BMI ≤ 18.50, (b) normal weight: BMI between 18.50 and 24.99, (c) overweight: BMI between 25.00 and 29.99, and (d) obesity: BMI ≥ 30.00. For this case, self-reported BMI was used—that is, this measure was based on the responses of the surveyed people about their weight and height. Self-perceived health, coffee and alcohol consumption during mandatory social isolation, smoking and consumption of psychoactive substances during mandatory social isolation were also retrieved.
- COVID-19 knowledge and preventive behaviors including: the number of days in mandatory social isolation; confinement level; COVID-19 information media; knowledge of those infected with COVID-19; self-perceived level of knowledge of COVID-19; self-perceived level of concern regarding COVID-19; following preventive measures for COVID-19; and use of preventive measures.

### 2.3. Statistical Analysis

Descriptive analysis was carried out for the qualitative and quantitative variables. The Kolmogorov–Smirnov test was used to verify a normality distribution in the main outcome and other quantitative variables. Furthermore, nonparametric tests were used: the Mann–Whitney U test for dichotomous variables and the Kruskal–Wallis test for polychotomous variables. Test reliability was obtained, by using Kuder-Richardson Formula 20, or KR-20 for the following variables: knowledge of those infected with COVID-19, self-perceived level of knowledge of COVID-19, self-perceived level of concern regarding COVID-19. In addition, a linear multivariate regression analysis was carried out to evaluate the simultaneous and reciprocal effect of the explanatory variables in the PSS-14. For that means, hierarchical regression models to see the change on the level of variance (R2) and accumulated variance. In this case, we used three models: Model 1 by adjusting for sociodemographic variables; Model 2 by adding health status variables, and Model 3 by adding level of knowledge and preventive behaviors variables. A logarithmic transformation was previously applied to the stress variable (PSS-14) because of the absence of a normal distribution. The study had a confidence level of 95%, and in all the tests, *p* < 0.05 was considered an indication of significance. Statistical analyses were performed using SPSS^®^ v. 25.0 (IBM, New York, NY, USA).

### 2.4. Ethics

The Ethical Committee of the Faculty of Dentistry at the University of Antioquia approved the study (Act 9-2020). Adhering to international standards for online surveys, all respondents of the survey completed an informed consent question on the first page of the questionnaire, and participants could reject or approve being involved in the study. Confidentiality was guaranteed throughout the research process in accordance with the Declaration of Helsinki.

## 3. Results

Initially there were 2442 responders of the survey, after the exclusion of inconsistent responses 2036 were used for the analysis. Table 1 shows the PSS-14 scores according to the sociodemographic characteristics of the sample. The mean PSS-14 score was 24.76 (±11.76), and a statistically significant association of perceived stress was found among variables such as age, sex, type of academic training, and place of origin (*p* < 0.05). Similarly, statistically significant differences were found in the PSS-14 scores and the variables: the number of people at home during mandatory social isolation (higher scores when the number is >3, *p* < 0.05); labor, academic, and home responsibilities during mandatory social isolation (higher scores when responsibilities increased, *p* < 0.001, *p* < 0.05, and *p* < 0.05, respectively); income level during mandatory social isolation (higher scores when income decreased, *p* < 0.05).

Table 2 shows the PSS-14 scores according to health variables and habits. Statistically significant differences were found in the case of BMI (higher score when underweight, *p* < 0.001), and self-perceived health (higher score when health is perceived as poor or very poor, *p* < 0.001). Also, higher PSS-14 scores and statistically significant differences were found when the consumption of coffee and psychoactive substances increased during mandatory social isolation (*p* < 0.001 in both cases).

Table 3 shows the perceived stress according to variables related to COVID-19 knowledge and preventive behaviors variables during mandatory social isolation measures. Statistically significant differences were found for the following variables: Self-perception of level of knowledge of COVID-19 (higher scores with lower knowledge level, *p* < 0.001), self-perception of level of concern regarding COVID-19 (higher scores with higher concern, *p* < 0.001); following preventive measures for COVID-19 (higher scores when adherence is lower, *p* < 0.001); and use of preventive measures (higher score in the case of no use, except in the case of the use of gloves (*p* < 0.209)). Regarding Kuder-Richardson 20 Test (KR-20), low reliability/consistency was found for the variables: knowledge of those infected with COVID-19 and self-perceived level of knowledge of COVID-19. It is important to consider that the percentage of participants that did not know people infected with covid-19 is high (a little more than 48%). Similarly, 51% of participants referred a low level of concern about COVID-19. These percentages could help to explain the reliability/consistency for these variables. In the case of the variable self-perceived level of knowledge of COVID-19, a high reliability/consistency was found. This could be explained since a low level of knowledge for this disease could increase the perceived stress for participants (Table 3). 

According to the multivariate linear regression models (Table 4), when model 1 is considered, a negative statistically significant factor for the PSS-14 score was found for the income level during mandatory social isolation (decreased income increased the PSS-14 score, *p*= 0.039). When health status variables were added (model 2), positive statistically significant factors for the PSS-14 score were found for the variables to have older adults under care during mandatory social isolation (to have older adults increased the PSS-14 score, *p*= 0.037), and self-perception of level of concern regarding COVID-19 (a higher level of concern increased the PSS-14 score, *p* = 0.003). Negative statistically significant factor for the PSS-14 score was found for self-perceived health (a poorer health status increased the PSS-14 score, *p* = 0.008). Finally, when all variables were included in the model 3 (by adding level of knowledge and preventive behaviors variables), positive statistically significant factors for the PSS-14 score were found for the variables self-perceived level of concern regarding COVID-19 (*p* = 0.001) and consumption of coffee during mandatory social isolation (an elevated consumption level increased the perceived stress score, *p* = 0.037). Model 2 and 3 were statistically significant with important changes in the R^2^, and a good adjusted for the variables included were observed. 

## 4. Discussion

The main findings of this study account for the impact of the COVID-19 pandemic, especially considering the mandatory isolation measures implemented by different governments, on stress perceived by the sample of dentists and dental students who participated in the study in the Latin America and Caribbean region. In addition, we identify the sociodemographic, COVID-19-related, and health variables associated with this perceived stress. The extreme risk of SARS-CoV-2 infection in the general population has required the adoption, on a global scale, of measures commissioned by the health authorities of different countries to minimize the rate of infection [3,33,34]. This situation has had a significant impact on the social, working, and everyday life of the general populace—especially health workers, of which dentists and dental students are no exception. In general terms, there is evidence of an association of stress experienced by dental staff and sociodemographic factors/variables directly related to the pandemic and the general health, and some consumption habits as a consequence of the social isolation conditions in the Latin America and Caribbean region. Complex interactions were observed when multivariate models were considered by adding sociodemographic, health status, and knowledge and behaviors variables. In general terms, this study contributes to improving the body of scientific literature on the impact of the COVID-19 pandemic in dental staff of this countries, since studies that take into account mental health indicators in this population are scarce.

An important element to consider when interpreting the results is related to the comparability of the findings regarding the questionnaires to evaluate mental health and/or perceived stress and the specific social and geographic contexts where the studies are carried out. Numerous psychometric scales have been developed to assess mental health in this period to understand the psychological burden posed by COVID-19 on different populations worldwide. For instance, a scoping review published in 2020 indicated 15 scales measuring COVID-19-associated mental health problems validated among diverse populations across the world [35]. Specifically, on indicators related to mental health in dental staff [14,15,16,17,18,19,20], six studies have evaluated stress [14,15,18,19,22,23] by using different psychometric scales and instruments, including the Depression, Anxiety, and Stress Scale-21 (DASS-21) [15,19], the Kessler’s Psychological Distress Scale K6 [18], GP-CORE [23], and open-ended questions about stress-related factors [14,22].

Among the sociodemographic factors analyzed, age had a significant impact on the increase in perceived stress in the sample of dentists and students from Latin America and the Caribbean (bivariate analysis), where a social gradient was observed (the older the participant, the lower the level of perceived stress). These findings are similar to those reported by previous studies involving dentists from China, India, Israel, Italy, the UK and Germany [18,19], although with results contrary to a study carried out in dental health-care workers (DHCWs) in Russia [15]. Although sociodemographic conditions and geographic contexts are different, these studies contribute to give us other analysis perspectives. Our result could be explained by a greater decrease in active social life, which is typical of younger individuals, although the same studies may show other trends when other indicators complementary to stress are analyzed by understanding specific conditions.

Females reported a higher level of stress (bivariate analysis); this finding is similar to those of studies involving dental staff [19], although another study did not report differences between males and females [15]. Some studies focused on other mental health indicators have exhibited gender differences—for example, a study with a cross-sectional sample of DHCWs from 19 countries using social media platforms reported higher anxiety in females [20]. The same situation was found in the case of studies carried out in Ecuador [27] and Turkey [22]. For women, the scientific literature has focused on family, domestic, and labor aspects that represent an additional load to the isolation measures due to the COVID-19 pandemic, as well as how this situation could affect mental health, especially in female health-care workers [36,37]. It is important to pay attention to the fact that specific studies in dental staff are still inconsistent, as they do not involve segmented analyses by sex to observe gender biases by including other social and contextual variables.

The COVID-19 pandemic experienced worldwide has resulted in several changes in people’s daily lives. For this reason, in this study, when specific elements related to the pandemic were asked, statistically significant differences were found in the bivariate analyses. For students, this meant new academic responsibilities mediated by remote and virtual strategies and some additional financial burdens in the context of great difficulties, among other circumstances [14,29]. In professionals, new labor adaptations were created, and this caused a decrease in working hours and even suspended face-to-face clinical activities [8,9,10,21]. Therefore, a decrease in income and an increase in domestic responsibilities were found [9,10].

Since the WHO declared the COVID-19 outbreak a pandemic in March 2020 [38], numerous dental practice modifications have been gradually implemented to adhere to international biosafety protocols and reduce the risk of infection [39,40]. In this sense, it is evident that measures adopted, such as hand hygiene, the use of masks, the suspension of activities with a high concentration of people, and social distancing, have triggered fear, anxiety, stress, and uncertainty mainly in health-care professionals, including dentists [10,11,17]. 

Although we did not find significant findings concerning perceived stress and the use of information media, significant differences in self-perceived stress scores were observed with the knowledge (bivariate) and concerns (bivariate/multivariate) about the COVID-19. In this sense, the impact of social media on the mental health of dental staff should be considered. A cross-sectional study conducted in DHCWs from 19 countries showed that the social media infodemic has affected the psychological well-being of dental students and dentists since a statistically significant association was found between the presence of anxiety and the frequency of use of social media [20]. However, we found variables associated in the bivariate analyses (statistically significant) with the stress perceived by the study’s participants and that are related to knowledge and attitudes related to COVID-19. Among these, we could mention, self-perceived level of knowledge of COVID-19, self-perceived level of concern regarding COVID-19. All these variables are explained by the daily aspects that dental staff live in the face of the pandemic, and that are in line with other studies [14,15,18,19,22,23].

Health as a biological and social process should be analyzed from a comprehensive and integrating perspective. In this study, a low self-perception of health was associated with the presence of greater stress in dentists and dental students in the region. The health situation of health workers, and especially their level of risk of infection, has been a subject of analysis in the scientific literature [37,41]. A study carried out in Germany found some differences between previous chronic conditions and the presence of indicators of poor mental health, such as stress, depression, and anxiety (although not statistically significant in most cases) [19]. The presence of comorbidities and other chronic conditions could affect general health and increase anxiety and stress in dental professionals. Further research could elucidate the relationship between mental and physical health in this population.

An association between an increase in the consumption of coffee (bivariate/multivariate analyses) and psychoactive substances (bivariate analysis) and an increase in stress level was found in the study participants. In the first case, some studies have evaluated the impact of COVID-19 on dietary habit changes [42,43] and the effects of fear and anxiety on nutrition in this pandemic period [44]. In our study, 29% of the participants increased coffee consumption. Furthermore, 418 people mentioned consuming psychoactive substances, and 10% of them mentioned an increase in their consumption habit. The relationship between loneliness, mental health, and substance abuse, especially in young adults, during the COVID-19 pandemic has been shown in a previous study conducted in the US [45].

Closely related to dietary habits and health situation is the BMI. In our sample, a third of the responders reported being overweight or obese, and 4% reported being underweight. However, the impact on perceived stress was higher in underweight individuals compared to the other groups, with statistically significant differences in the PSS-14 scores. A cross-sectional study in the general Jordanian population revealed negative changes in healthy nutritional behavior during the COVID-19 isolation measures, and this situation resulted in an increase in body weight [13]. Further research is required to assess the nutritional status of dental students and dentists through the use of other indicators.

Although we have discussed previously how some trends and differences occurred in bivariate analyses, it is important to consider the interactions that occurred between the different study variables. That situation was influenced when multivariate analyses were performed by using different linear (hierarchical) regression models. The reality of the Latin American and Caribbean region is complex and diverse and has different social, economic, and political contexts and therefore the impact of the COVID-19 pandemic exhibits different patterns of analysis. However, the health of individuals in different dimensions was affected and this explains the significance of variables such as self-perception of health and the level of concern for the pandemic. Also, coffee consumption, which is very characteristic of the region was associated with the level of stress perceived by the participants. These factors can intervene in social factors related to personal, academic, and labor life, as was discussed.

It is important to comment on the strengths and limitations of this research. The strengths include how the information collection instruments were carefully developed and tested through a pilot test and, in some cases, internationally validated. The findings are comparable with the literature despite the fact that exist multiplicity of faces and features regarding the impact of pandemic in specific territories, social groups and professional staffs. The sample included participants from 21 countries in the Latin American and Caribbean region—which allows recognition of their own social and health characteristics, and we obtained a high cooperation rate (83.4%). As limitations, we should consider that the geographic representation was not homogeneous and is not statistically representative. The cross-sectional nature of the study does not allow for causal inferences. The survey was carried out during the initial period of the pandemic; hence, some of the factors studied could show some variation. We can not discard possible sources of information bias, but this is a common situation when digital or online surveys are conducted. Conversely, it is necessary to establish specific analyses by region and make comparisons between countries since the compulsory social isolation measures were not implemented under equal conditions. Finally, we found a low level of consistency (KR-20 = 0.332) for the variable self-perceived level of concern regarding COVID-19, and these findings should be cautiously interpreted. Factors regarding the type of sample, the influence of the pandemic itself, and the period of fieldwork could affect this consistency.

Accepting the aforementioned limitations, this study constitutes an important input for understanding the mental health situation of dentists and dental students in Latin America and the Caribbean. We intend to continue this research through follow-up strategies that allow evaluation of the conditions of this population in other phases of the pandemic, especially considering the expectations regarding mass vaccination of both health personnel and the general population. 

New methodological approaches, qualitative and mixed, will enable approaching this social phenomenon from different points of view, as well as help to understand the reality experienced in specific contexts, including other social, labor, and health (physical, mental and psychosocial) indicators. There is evidence of the need for policies and strategies based on social reality for each country and a global level that contribute to mitigating the possible consequences of the pandemic on the social and health situation of the professional and student dental population. Epidemiological monitoring and evaluation systems that facilitate obtaining reliable data on indicators of physical, mental, and psychosocial health of this population, considering this pandemic, should also be established.

## 5. Conclusions

The present study evidenced the short-term impact of the COVID-19 pandemic and, the mandatory isolation measures imposed as a direct consequence of the pandemic on the mental health situation, expressed in the level of stress perceived in dentists and dental students of Latin America and the Caribbean. Some factors were directly associated with stress, such as sociodemographic variables, specific situations experienced during the pandemic, the state of health, and some habits of consumption of substances. Multivariate hierarchical regression models showed the complex interactions among sociodemographic, health and knowledge, and preventive behaviors during the pandemic. This reflects the diverse conditions in the region that need further epidemiological surveillance through additional research.

## Figures and Tables

**Table 1:** Perceived stress according to sociodemographic variables during mandatory social isolation measures in the study sample (*n* = 2036).

Variables	*n*	%	Perceived Stress (PSS-14 Score)		
			X	SD	*p*-Value
Age					
Mean (SD)	32.69	11.76			
18–24	606	29.76	26.69	7.30	<0.001 *
25–34	749	36.79	25.30	7.10	
≥35	681	33.45	22.44	7.61	
Sex					
Male	603	29.62	23.33	7.49	<0.001 **
Female	1433	70.38	25.36	7.47	
Type of academic training					
Dental student	724	35.56	26.81	7.20	<0.001 **
Dentist	1312	64.44	23.62	7.47	
Specialty					
Yes	913	69.64	23.20	7.59	0.002 **
No	398	30.36	24.62	7.13	
Place of origin					
Mexico, Central America and the Caribbean	164	8.06	23.24	8.01	0.005 **
South America	1872	91.94	24.89	7.48	
Number of people at home during social isolation measures (Mean, DS)					
≤3	1395	68.55	24.43	7.50	0.004 *
>3	640	31.45	25.48	7.56	
Children under care during social isolation measures					
No	1366	67.09	24.76	7.54	0.854 *
Yes	670	32.91	24.75	7.53	
Older adults under care during social isolation measures					
No	1450	71.22	24.64	7.49	0.318 *
Yes	586	28.78	25.06	7.62	
Labor responsibilities during social isolation measures					
Decreased	707	43.43	23.52	7.61	<0.001 **
Equal	323	19.84	24.34	7.01	
Increased	598	36.73	25.28	7.22	
Academic responsibilities during social isolation measures					
Decreased	420	24.50	24.73	7.57	0.014 **
Equal	418	24.39	24.20	7.02	
Increased	876	51.11	25.35	7.67	
Domestic (home) responsibilities during social isolation measures					
Decreased	21	1.04	26.00	6.03	0.021 **
Equal	512	25.36	24.05	7.71	
Increased	1486	73.60	25.04	7.46	
Income level during social isolation measures					
Decreased	1129	67.52	24.86	7.61	0.033 **
Equal	469	28.05	23.68	6.98	
Increased	74	4.43	24.84	7.23	
All	2036	100.00	24.76	7.53	

* Kruskal Wallis Test. ** Mann Whitney U Test. Responses with missing values (Specialty: Responses only for dentists and missing values = 1; Number of people at home during social isolation measures: *n* = 1; Domestic (home) responsibilities during social isolation measures: *n* = 17).

**Table 2:** Perceived stress according to health variables and habits during mandatory social isolation measures in the study sample (*n* = 2036).

Variables	*n*	%	Perceived Stress (PSS-14 Score)		
			Mean	SD	*p*-Value
BMI					
Underweight	83	4.17	27.33	8.10	<0.001 **
Normal	1242	62.41	25.11	7.48	
Overweight	531	26.68	23.91	7.38	
Obesity	134	6.73	23.40	7.95	
Self-perceived health					
Very poor	6	0.29	37.50	7.34	<0.001 **
Poor	25	1.23	33.56	7.88	
Fair	299	14.69	28.15	7.08	
Good	1263	62.03	24.59	7.01	
Excellent	443	21.76	22.27	7.90	
Coffee consumption during mandatory social isolation (*n* = 1601)					
Decreased	333	20.80	24.78	7.85	<0.001 **
Equal	806	50.34	24.04	7.27	
Increased	462	28.86	26.46	7.27	
Alcohol consumption during mandatory social isolation (*n* = 1256)					
Decreased	817	65.05	24.95	7.18	0.308 **
Equal	285	22.69	24.26	7.71	
Increased	154	12.26	25.90	7.88	
Smoking during mandatory social isolation (*n* = 494)					
Decreased	346	70.04	25.61	6.93	0.263 **
Equal	97	19.64	24.25	7.19	
Increased	51	10.32	26.02	7.53	
Consumption of psychoactive substances during mandatory social isolation (*n* = 418)					
Decreased	262	62.68	24.65	6.93	<0.001 **
Equal	116	27.75	25.86	7.31	
Increased	40	9.57	30.78	7.52	

** Kruskal Wallis Test. Responses with missing values (BMI: *n* = 46).

**Table 3:** Perceived stress according to stress according to COVID-19 knowledge and preventive behaviors variables during mandatory social isolation measures in the study sample (*n* = 2036).

Variables	*n*	%	Perceived Stress (PSS-14 Score)		
			Mean	SD	*p*-Value
Number of days in mandatory social isolation (Mean, DS)					
			(60.97	25.88)	
≤60	1119	54.96	24.86	7.24	0.401 *
>60	917	45.04	24.63	7.88	
Confinement level					
I have not gone out any day	214	10.51	26.02	7.54	0.080 **
I have gone out very little	1614	79.27	24.61	7.55	
I have been out frequently	117	5.75	24.62	7.54	
I’ve been out every day	91	4.47	24.58	6.98	
COVID-19 Information Media					
Traditional	61	3.00	24.93	8.44	0.941 *
Virtual	1975	97.00	24.75	7.50	
Knowledge of those infected with COVID-19 ***					
No	1050	51.57	24.50	7.58	0.109 *
Yes	986	48.43	25.03	7.48	
Self-perceived level of knowledge of COVID-19 ***					
Low	1618	79.47	25.10	7.36	<0.001 *
High	418	20.53	23.45	8.03	
Self-perceived level of concern regarding COVID-19 **					
Low	1036	50.88	23.71	7.68	<0.001 *
High	1000	49.12	25.84	7.22	
Following of preventive measures for COVID-19					
Never	2	0.10	29.00	0.00	<0.001 **
Rarely	6	0.29	23.50	9.16	
Usually	561	27.55	25.88	6.83	
Sometimes	47	2.31	27.64	7.95	
Always	1420	69.74	24.22	7.71	
Use of preventive measures					
Face mask					
No	35	1.72	27.66	5.60	0.021 *
Yes	2001	98.28	24.71	7.55	
Hand washing					
No	23	1.13	29.48	6.89	0.007 *
Yes	2013	98.87	24.70	7.52	
Social distancing					
No	152	7.47	26.43	6.53	0.004 *
Yes	1884	92.53	24.62	7.59	
Disinfection protocol at the entrance of the house					
No	353	17.34	25.80	7.95	0.004 *
Yes	1683	82.66	24.54	7.42	
Covering up when coughing or sneezing					
No	185	9.09	25.64	7.05	0.035 *
Yes	1851	90.91	24.67	7.57	
Care measures for the elderly and risk personnel					
No	756	37.13	25.10	7.17	0.043 *
Yes	1280	62.87	24.55	7.73	
Avoid shaking hands or kissing					
No	188	9.23	26.11	7.68	0.005 *
Yes	1848	90.77	24.62	7.50	
Use of gloves					
No	1289	63.31	24.88	7.69	0.209 *
Yes	747	36.69	24.54	7.26	
Use of face masks, hand washing and social distancing					
No	180	8.84	26.82	6.62	<0.001 *
Yes	1856	91.16	24.56	7.59	
Number of preventive measures for COVID-19			6.54	1.31	
≤7	1543	75.79	24.96	7.52	0.013 *
>7	493	24.21	24.13	7.54	

* Mann Whitney U Test. ** Kruskal Wallis Test. *** Kuder-Richardson 20 Test (KR-20) for the following variables: Knowledge of those infected with COVID-19 (KR-20 = 0.079), Self-perceived level of knowledge of COVID-19 (KR-20 = 0.782), Self-perceived level of concern regarding COVID-19 (KR-20 = 0.332).

**Table 4:** Hierarchical multiple lineal regression models for the perceived stress scores in the study sample (*n* = 2036).

Variables	Determination Coefficient % (R2%)	Change of R2%	*p*-Value Change of R2%	Constant	Non-Standardized Regression Coefficient	Standardized Regression Coefficient	Confidence Interval 95%	*p*-Value	*p*-Value Model
Model 1									
Sociodemographic variables									
Age	0.75	7.50	0.315	20.665	−1.323	−0.106	−3.303–0.658	0.189	0.315
Sex					0.875	0.055	−1.592–3.342	0.485	
Type of academic training					−1.721	−0.106	−2.994–0.447	0.080	
Specialty					−0.311	−0.020	−2.700–2.078	0.797	
Place of origin					2.437	0.087	−1.908–6.782	0.270	
Number of people at home during mandatory social isolation					1.529	0.095	−1.149–4.202	0.260	
Children under care during mandatory social isolation					−0.948	−0.064	−3.361–1.466	0.439	
Older adults under care during mandatory social isolation					2.201	0.135	−0.415–4.818	0.099	
Labor responsibilities during mandatory social isolation					0.660	0.071	−1.263–2.582	0.499	
Academic responsibilities during mandatory social isolation					−0.374	−0.036	−2.309–1.561	0.703	
Domestic (home) responsibilities during mandatory social isolation					1.379	0.079	−1.310–4.067	0.313	
Income level during mandatory social isolation					−2.408	−0.192	−4.689–−0.127	0.039	
Model 2									
Sociodemographic variables									
Age	20.30	12.80	0.002	25.426	−0.857	−0.069	−2.782–1.067	0.380	0.007
Sex					0.351	0.022	−2.116–2.817	0.779	
Type of academic training					−1.717	−0.105	−2.991–0.442	0.081	
Specialty					−0.182	−0.012	−2.499–2.135	0.877	
Place of origin					0.837	0.030	−3.423–5.097	0.698	
Number of people at home during mandatory social isolation					0.845	0.052	−1.782–3.471	0.526	
Children under care during mandatory social isolation					−0.761	−0.051	−3.091–1.569	0.520	
Older adults under care during mandatory social isolation					2.688	0.165	0.169–5.207	0.037	
Labor responsibilities during mandatory social isolation					0.148	0.016	−1.726–2.022	0.876	
Academic responsibilities during mandatory social isolation					−0.625	−0.060	−2.568–1.319	0.526	
Domestic (home) responsibilities during mandatory social isolation					0.257	0.015	−2.415–2.929	0.850	
Income level during mandatory social isolation					−1.305	−0.104	−3.543–0.933	0.251	
Health status variables									
BMI					−0.397	−0.038	−2.039–1.244	0.633	
Self-perceived level of concern regarding COVID-19					3.213	0.224	1.107–5.318	0.003	
Self-perceived health					−2.557	−0.218	−4.439–−0.676	0.008	
Coffee consumption during mandatory social isolation					1.053	0.108	−0.542–2.649	0.194	
Alcohol consumption during mandatory social isolation					0.727	0.069	−1.199–2.652	0.457	
Smoking during mandatory social isolation					−1.316	−0.115	−3.817–1.185	0.300	
Consumption of psychoactive substances during mandatory social isolation					0.766	0.060	−2.134–3.666	0.602	
Model 3									
Sociodemographic variables									
Age	32.40	16.10	0.070	56.252	−0.415	−0.033	−2.530–1.700	0.699	0.003
Sex					0.630	0.040	−2.001–3.260	0.637	
Type of academic training					−1.590	−0.098	−2.868–0.311	0.150	
Specialty					−0.297	−0.019	−2.673–2.080	0.805	
Place of origin					0.346	0.012	−3.912–4.604	0.873	
Number of people at home during mandatory social isolation					0.465	0.029	−2.244–3.173	0.735	
Children under care during mandatory social isolation					−1.169	−0.079	−3.582–1.245	0.340	
Older adults under care during mandatory social isolation					2.471	0.152	−0.158–5.099	0.065	
Labor responsibilities during mandatory social isolation					−0.248	−0.027	−2.196–1.700	0.802	
Academic responsibilities during mandatory social isolation					−0.250	−0.024	−2.226–1.726	0.803	
Domestic (home) responsibilities during mandatory social isolation					−0.660	−0.038	−3.383–2.063	0.632	
Income level during mandatory social isolation					−1.383	−0.110	−3.645–0.879	0.229	
Health status variables									
BMI					−0.261	−0.025	−1.964–1.442	0.762	
Self-perceived level of concern regarding COVID-19					3.889	0.271	1.656–6.122	0.001	
Self-perceived health					−2.564	−0.219	−4.491–−0.637	0.009	
Coffee consumption during mandatory social isolation					1.797	0.184	0.114–3.479	0.037	
Alcohol consumption during mandatory social isolation					0.721	0.068	−1.180–2.623	0.454	
Smoking during mandatory social isolation					−1.723	−0.151	−4.307–0.862	0.190	
Consumption of psychoactive substances during mandatory social isolation					0.164	0.013	−2.727–3.055	0.911	
Knowledge and preventive behaviors variables									
Number of days in mandatory social isolation					1.376	0.093	−0.913–3.665	0.237	
Confinement level					10.386	0.121	−0.448–3.220	0.137	
COVID-19 Information Media					−4.900	−0.115	−11.484–1.684	0.143	
Knowledge of those infected with COVID-19					−2.245	−0.157	−4.566–0.077	0.058	
Self-perceived level of knowledge of COVID-19					0.709	0.043	−1.848–3.265	0.584	
Following of preventive measures for COVID-19					−0.428	−0.045	−2.089–1.234	0.612	
Face mask					1.404	0.015	−14.328–17.137	0.860	
Hand washing					−15.324	−0.163	−31.188–0.540	0.058	
Social distancing					−2.976	−0.088	−9.387–3.435	0.360	
Disinfection protocol at the entrance of the house					−0.740	−0.036	−4.323–2.844	0.684	
Covering up when coughing or sneezing					−0.854	−0.038	−4.681–2.972	0.660	
Care measures for the elderly and risk personnel					−2.225	−0.140	−5.034–0.584	0.120	
Avoid shaking hands or kissing					−0.646	−0.021	−6.059–4.767	0.814	
Use of gloves					−0.894	−0.062	−5.005–3.217	0.668	
Use of face masks, hand washing and social distancing					0.680	0.113	−8.319–13.076	0.662	
Number of preventive measures for COVID-19					1.715	0.113	−2.804–6.234	0.454	
